# The choice of null distributions for detecting gene-gene interactions in genome-wide association studies

**DOI:** 10.1186/1471-2105-12-S1-S26

**Published:** 2011-02-15

**Authors:** Can Yang, Xiang Wan, Zengyou He, Qiang Yang, Hong Xue, Weichuan Yu

**Affiliations:** 1 Department of Electronic and Computer Engineering, Hong Kong University of Science and Technology, Hong Kong; 2 School of Software, Dalian University of Technology, China; 3 Department of Computer Science, Hong Kong University of Science and Technology, Hong Kong; 4 Department of Biochemistry, Hong Kong University of Science and Technology, Hong Kong

## Abstract

**Background:**

In genome-wide association studies (GWAS), the number of single-nucleotide polymorphisms (SNPs) typically ranges between 500,000 and 1,000,000. Accordingly, detecting gene-gene interactions in GWAS is computationally challenging because it involves hundreds of billions of SNP pairs. Stage-wise strategies are often used to overcome the computational difficulty. In the first stage, fast screening methods (e.g. Tuning ReliefF) are applied to reduce the whole SNP set to a small subset. In the second stage, sophisticated modeling methods (e.g., multifactor-dimensionality reduction (MDR)) are applied to the subset of SNPs to identify interesting interaction models and the corresponding interaction patterns. In the third stage, the significance of the identified interaction patterns is evaluated by hypothesis testing.

**Results:**

In this paper, we show that this stage-wise strategy could be problematic in controlling the false positive rate if the null distribution is not appropriately chosen. This is because screening and modeling may change the null distribution used in hypothesis testing. In our simulation study, we use some popular screening methods and the popular modeling method MDR as examples to show the effect of the inappropriate choice of null distributions. To choose appropriate null distributions, we suggest to use the permutation test or testing on the independent data set. We demonstrate their performance using synthetic data and a real genome wide data set from an Aged-related Macular Degeneration (AMD) study.

**Conclusions:**

The permutation test or testing on the independent data set can help choosing appropriate null distributions in hypothesis testing, which provides more reliable results in practice.

## Background

Single-nucleotide polymorphisms (SNPs) serve as markers for mapping disease-associated genetic variants. It has been well known that SNP profiles are associated with a variety of diseases [1]. High-throughput genotyping technologies have been used to assay hundreds of thousands of SNPs in the human genome. Many single-locus based methods [2] have been proposed and many susceptibility determinants have been identified [1]. However, these identified SNPs seem to be insufficient in explaining the genetic contributions to complex diseases [3]. Researchers start to suspect that the causality of many common disease are more related with gene-gene interactions rather than with single genetic variations [3,4]. For many common complex diseases, some SNPs have shown little main effects while their interactions are significantly associated with disease traits [5-7]. Consequently, detecting gene-gene interactions is a topic of current interest in GWAS [4]. Many methods have recently been proposed to identify interaction patterns associated with diseases, including MDR [6], CPM [5], RPM [8], BGTA [9], SNPRuler [10], LASSO [11-13], HapForest [14], BOOST [15], PLINK [16], BEAM [17], SNPHarvester [18] and INTERSNP [19]. However, a key issue of applying most of these methods in GWAS is the computational burden [4]. For example, to find pairwise interactions from 500,000 SNPs, we need 1.25 × 10^11^ statistical tests in total. To address this issue, screening approaches [20] have been proposed. The whole process of detecting gene-gene interactions is then divided into three stages:

• Screening: Evaluate the importance of each SNP and assign it a score. Those SNPs with scores lower than the given threshold are removed without further consideration. Often a small portion of SNPs remains. This stage is often accomplished by fast algorithms using heuristics to reduce the search space of the next stage. One example is the popular screening method Tuning ReliefF [21].

• Modeling: Search for the best combination of SNPs in the remaining SNPs. The exhaustive search can be used in this stage because the number of remaining SNPs is small, e.g., the popular modeling methods MDR and CPM. During the search process, the importance of a SNP combination is often measured by its prediction accuracy (typically evaluated by cross-validation). Thus, the best SNP combination and its corresponding interaction pattern can be identified in term of prediction accuracy.

• Testing: Assess the significance of interaction patterns by hypothesis testing.

**Figure 1:**
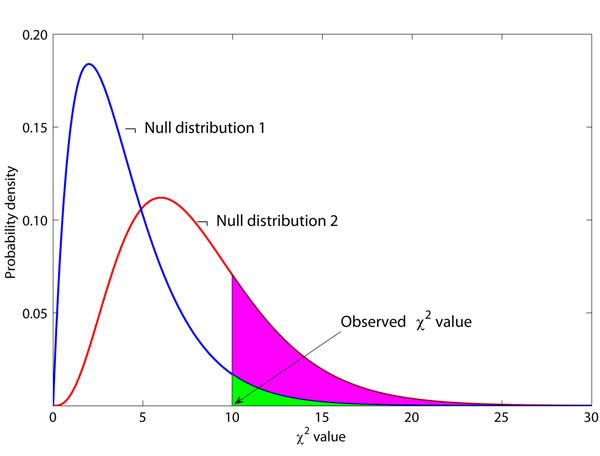
**A toy example illustrating the effect of the inappropriate choice of null distributions.**  Null distribution 1 follows the  and null distribution 2 follows the . The observed *χ^2^* value is 10, and the *P*-values are 0.0404 and 0.2650 for these two null distributions, respectively. Suppose *P* = 0.05 is the threshold of hypothesis testing. Then *P* = 0.0404 indicates a significant result, while *P* = 0.2650 does not. If the true null distribution is , then the use of  will give many false positive results.

Hypothesis testing employed in the testing stage is also referred to as “feature assessment” in [22]. A critical issue in feature assessment is to choose an appropriate null distribution for hypothesis testing. An inappropriate null distribution may lead to an over-optimistic result (high false positive error) or an over-conservative result (high false negative error) [23]. Figure 1 gives a toy example. In this figure, null distribution 1 follows the *χ*^2^ distribution with the degree of freedom *df* = 4, denoted as , and null distribution 2 follows . If the true null distribution is , then using  for hypothesis testing will give many false positive results.

In this paper, we show through simulations that both screening and modeling may change the null distribution used in hypothesis testing. However, many methods (such as MDR combined with Tuning ReliefF [6,21] and some other stage-wise methods [24]) neglect the rightward shift of the original null distribution (as illustrated in Figure 1) caused by screening and modeling. They inevitably suffer from the higher false positive rate. We have also noticed that some methods [25-27] modify the test statistics, which causes the leftward shift of the original null distribution. If we still stick to the theoretical null distribution in hypothesis testing, we may produce conservative results (see the discussion section). To address this issue, we suggest to use the permutation test and testing on the independent data set. The permutation test uses the re-sampling method to estimate the changed null distribution for hypothesis testing. Testing on the independent data set can reserve the theoretical null distribution. Through simulation experiments and the experiment on a real genome-wide data set from an AMD study, we demonstrate that the appropriate choice of null distributions leads to more reliable results.

## Results

### Simulation study of the inappropriate choice of null distributions

The huge number of SNPs in GWAS poses a heavy computational burden for detecting gene-gene interactions. The exhaustive search of all pairwise interactions and further using cross-validation to evaluate them (e.g. MDR [6]) become impractical in GWAS. To make it computationally feasible, a screening method is applied to the whole data set to pre-select a small subset of SNPs. Then the exhaustive search can be applied to identify the most likely disease-associated SNPs. At last, hypothesis testing is conducted on identified SNPs. The importance of hypothesis testing is briefly discussed in the discussion section. Here we use MDR and some efficient screening methods [21,28-30] as examples to show that null distributions are affected by these methods Throughout this paper, we use the latest MDR software (MDR 2.0 beta 8.1) to perform all experiments. It also implements various screening methods, such as ReliefF, Tuning ReliefF, and SURFSTAR. We first show that the modeling process of MDR changes the null distribution in its search process. Then we show that MDR coupled with some screening methods further changes the null distribution.

#### The null distribution of MDR

MDR is a popular non-parametric approach for detecting all possible *k*-way (*k* = 2,…, d) combinations of SNPs that interact to influence disease traits. MDR runs 10-fold cross-validations, and uses the prediction errors and the consistencies to search for the optimal set of *k*-way interactions. For each of the selected *k*-way interactions, MDR constructs a 2 × 2 contingency table by partitioning the samples into the high-risk and low-risk groups. Then MDR conducts hypothesis testing based on this 2 × 2 contingency table. Table 1 illustrates how this is done by MDR. The authors claimed that MDR could reduce the *k* dimensional model into a 1 dimensional model [6]. Therefore, the current MDR software conducts the statistical test on a 2 × 2 contingency table using  as its null distribution. This statement is biased because the higher dimensional space has to be browsed in order to construct the 2 × 2 continency table [31]. Here we use simulation experiments to show the correct null distribution in hypothesis testing for MDR.

**Table 1:** A toy example illustrating how MDR collapses two 3 × 3 genotype tables to a 2 × 2 contingency table

Case	*BB*	*Bb*	*bb*	Control	*BB*	*Bb*	*bb*	MDR table	Case	Control
*AA*	179	119	**18**	*AA*	199	126	*15*			
*Aa*	**315**	**173**	**26**	*Aa*	*306*	*164*	*17*	Low-risk	399	443
*aa*	101	59	**10**	*aa*	118	*49*	*6*	High-risk	**601**	*557*

Suppose the threshold of the odds ratio in MDR is specified to be *τ_OR_* = 1. Then the 2 × 2 contingency table is obtained by MDR in the following way: For the cells whose odd-ratio is higher than *τ_OR_* will be considered as high-risk. For case genotypes, the bold numbers indicate these genotypes are considered as high-risk. Their summation equals to 601 as shown in bold font in the MDR table. For control genotypes, the italic numbers also indicate these genotypes are considered as high-risk. Their summation equals to 557 as shown in italic font in the MDR table. For the low-risk cells, this is done in the same way. Statistical tests can be conducted based on the 2 × 2 contingency table: χ^2^ = 3.9711 for the Pearson χ^2^ test and χ^2^ = 3.9726 for the likelihood ratio test.

We design two scenarios with three settings of *d*-way interactions (*d* = 2, 3, 4), one showing the true null distributions of MDR (without search) and another showing the change of null distributions of MDR (with search). For each scenario of this experiment, we generate 500 null data sets, each of which contains 2,000 samples.

**Figure 2:**
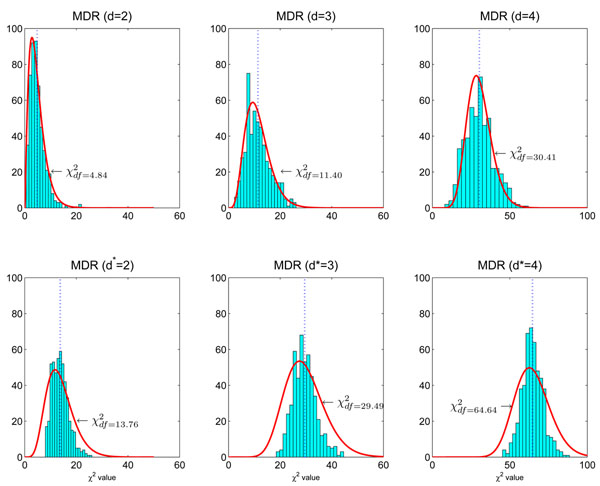
**Null distributions affected by MDR modeling.** The null distributions are estimated using 500 simulated null data sets. Each null data set contains *n* = 2000 samples. **Upper panel:** From left to right, each data set has ***L*** = 2, ***L*** = 3, ***L*** = 4 SNPs. MDR can be applied to these data sets without model search to fit the two-factor model (*d* = 2), the three-factor model (*d* = 3), and the four-factor model (*d* = 4). The resulting null distributions follows *χ*^2^ distributions with *df* = 4.84, 11.40, 30.41, respectively. **Lower panel:** Each null data set contains *n* = 2000 samples and ***L*** = 20 SNPs. MDR is directly applied to each data set. MDR searches all possible models and cross-validation is used to assess each model. The best two-factor model (d* = 2), the best three-factor model (d* = 3), and the best four-factor model (d* = 4) are identified. Their distributions, shown from left to right, do not strictly follow *χ*^2^ distributions.

In the first scenario, we generate data sets containing two, three and four SNPs for the settings *d* = 2, 3, 4, respectively. All SNPs are generated using the Hardy-Weinberg principle with minor allele frequencies uniformly distributed in [0.05,0.5]. By doing so, the MDR model can be directly fitted without search. Let us take *d* = 2 as an example. For each null data set, we first obtain a genotype contingency table as shown in Table 1, and then collapse it into a 2 × 2 contingency table. Next we conduct the statistical test (either the Pearson *χ*^2^ test or the likelihood ratio test can be used since their difference is ignorable). The histogram of the statistics forms the null distribution. For *d* = 3,4, this can be done in the same way. The histograms of these null distributions obtained from 500 null data sets are shown in the upper panel of Figure 2. We observe that the null distributions of MDR (without search) follow the *χ*^2^ distributions. The estimated degrees of freedom of the *χ*^2^ distributions are *df* = 4.84, *df* = 11.40 and *df* = 30.41 for *d* = 2, *d* = 3 and *d* = 4, respectively. The non-interger degree of freedom is well defined, see [31-33]. This clearly indicates that  is not an appropriate null distribution for MDR.

In the second scenario, we generate the data sets containing 20 SNPs for all three cases. These SNPs are generated in the same way as in the first scenario. In this scenario, MDR first searches for the best d*-way interactions using 10-fold cross-validation and then conducts the testing. The results are shown in the lower panel of Figure 2. Compared with the first scenario, the null distributions change when MDR searches for the best d*-way interactions (d* = 2, 3,4) in *d* = 20 dimensions. We see that the null distributions do not strictly follow *χ*^2^ distributions. We use *χ*^2^ distributions to approximate them, and obtain their degrees of freedom as *df* = 13.76, *df* = 29.49 and *df* = 64.64, respectively.

In summary, our result shows the following facts:

• The null distribution in MDR does not follow  even when search process is not involved.

• The null distribution in MDR further changes when the search process is involved.

Therefore, hypothesis testing using  as the null distribution in MDR will give many false positive results.

#### The null distribution for MDR combined with screening methods

MDR works well for small studies with 100 or less SNPs. In GWAS, it is not practical to use this exhaustive search method. Therefore, many screening methods have been proposed to reduce the number of SNPs before MDR is applied to detect d*-way interactions. These screening methods include ReliefF [28,29], Tuning ReliefF (TURF) [21], SURF [34], SURFSTAR [30]. The reader is referred to [20] for a recent review on these screening methods.

**Figure 3:**
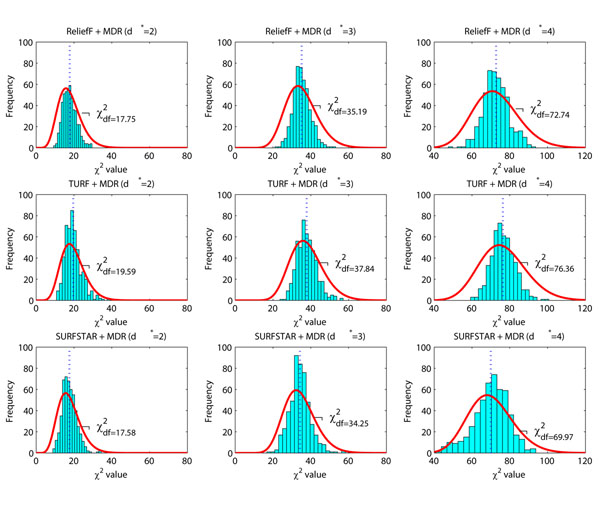
**Null distributions affected by screening methods.** The null distributions are estimated using 500 simulated null data sets. The null distributions shown in the lower panel of Figure 2, serve as the reference distributions (*df* = 13.76 for d* = 2, *df* = 29.49 for d* = 3 and *df* = 64.64 for d* = 4). The screening methods ReliefF, TURF and SURFSTAR are used to reduce the number of SNPs from ***L*** = 2000 to *d* = 20. For the remaining *d* = 20 SNPs, MDR is used to identify the best d*-way interactions (d* = 2, 3, 4). The resulting null distributions of these models, shown from left to right, do not strictly follow the *χ^2^* distribution. The null distributions shift rightwards, compared with those distributions in the lower panel of Figure 2.

The issue here is that after these screening methods are applied, the null distributions are further changed. To show the effect of screening methods on the null distribution, we generate 500 null data sets. Each data set contains 2,000 samples and each sample contains ***L*** = 2,000 SNPs. All SNPs are generated using the Hardy-Weinberg principle with minor allele frequencies uniformly distributed in [0.05,0.5]. Different screening methods, such as ReliefF, TURF, SURFSTAR, are first applied to reduce ***L*** = 2,000 SNPs to *d* = 20 SNPs. After that, MDR is applied to find the best d*-way interactions (d* = 2, 3, 4). Figure 3 presents the experiment results. The lower panel of Figure 2 serves as the reference distributions. It is obvious that all three screening methods further change the null distributions.

### Simulation study of the suggested solutions

**Figure 4:**
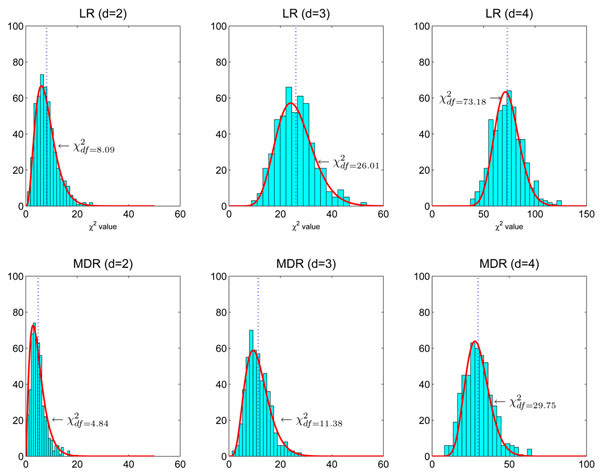
**Null distributions of testing on the independent data set.** We generate 500 null data sets. Each data set has 2000 samples and 2000 SNPs. We divide each data set into three subsets with nearly equal size. The first one is used for screening, the second one is for modeling and the third one is for hypothesis testing. **The upper panel**: Logistic regression (LR) is used in modeling. The degrees of freedom of the theoretical null distributions are *df* = 8,26,80 for 2,3,4-way interaction models, respectively. We see that the null distributions of LR match the theoretical null distributions well for 2,3-way interaction models. The resulting null distribution of the 4-way interaction model follows  Here *df* = 73.18 is smaller than the theoretical one (*df* = 80) because there are only about 666 samples in hypothesis testing. The number of samples is too small to accurately estimate the large degree of freedom of the theoretical null distribution (*df* = 80). **The lower panel:** MDR is used in modeling. We can see that the obtained null distributions are roughly the same with those shown in the upper panel of Figure 2.

Two solutions are suggested in the method section. The first one is the permutation test and the second is testing on the independent data set. The permutation test works well to estimate null distributions in practice. Although the computational cost of this method is high, it is still feasible to be applied in GWAS when an efficient screening method is available. It is unnecessary to show its performance in simulation studies since it is a standard way of calibrating the null distribution in hypothesis testing [35,36]. We demonstrate its performance using a real genome-wide data set from an AMD study in the next section. Here we show that the null distribution does not change when testing on an independent data set. We generate 500 null data sets. Each data set contains 2,000 samples and each sample has ***L*** = 2,000 SNPs. Each data set is partitioned into three subsets as nearly equal as possible: ***D***^(1)^, ***D***^(2)^, ***D***^(3)^. The screening method ReliefF is first applied to ***D***^(1)^, and the number of SNPs is reduced from ***L*** = 2,000 to *d* = 20 SNPs. The indices of the remaining 20 SNPs are collected in ***A***_1_. After that, stepwise logistic regression (LR) is applied to ***D***^(2)^ to find the best d*-way interactions (d* = 2, 3, 4) among the SNPs in ***A***_1_. At last, the likelihood ratio test is applied to the identified SNP set in hypothesis testing: it uses the difference between the deviance of the full d*-way logistic regression interaction model and the deviance of the null logistic regression model. Here we first use LR rather than MDR, because the null distributions of LR are known analytically. For 2, 3, 4-way full logistic regression interaction models, the null distributions follow *χ*^2^ distributions with *df* = 8, 26, 80, respectively. The experiment results are present in the upper panel of Figure 4. We can see that the obtained null distributions of logistic regression models match the theoretical null distributions well for 2, 3-way interaction models. For the 4-way interaction model, the estimated null distribution has a smaller degree of freedom than the theoretical null distribution. This is because about 666 samples are not enough to accurately estimate the large degree of freedom of the theoretical null distribution (*df* = 80). This is a disadvantage of using an independent data set for testing. When there are not enough samples, testing on an independent data set may have a lower power if its theoretical null distribution is used (This is an opposite case of the one shown in Figure 1).

To see the effect of MDR using the independent data set in hypothesis testing, we similarly divide each null data set into three subsets: ***D***^(1)^, ***D***^(2)^, ***D***^(3)^. ReliefF is applied to ***D***^(1)^ and then MDR is applied to ***D***^(2)^. At last, *χ*^2^ tests are conducted on ***D***^(3)^ using the 2 × 2 contingency tables obtained by the MDR method. The result is shown in the lower panel of Figure 4. We can see that the obtained null distributions of MDR agree with the distributions shown in the upper panel of Figure 2. This result clearly shows that the null distributions are not changed by screening and modeling when testing on the independent data set.

### Real data analysis using the suggested solutions: an experiment on the Aged-related Macular Degeneration (AMD) data set

We use the AMD data from [37] as a real example. The AMD study genotyped 116,204 SNPs on 96 cases and 50 controls. After applying quality control to the AMD data set, we have 82,143 qualified SNPs. Two significant loci, rs380390 and rs1329428, were reported in [37] based on the allelic association test with degree of freedom *df* = 1.

First, we use the latest MDR software to analyze this data set. ReliefF is applied to reduce the number of SNPs in AMD data from ***L*** = 82, 143 to *d* = 20. After that, MDR is applied to the remaining 20 SNPs to search for the best model. The result is given in Table 2. The AMD data set is available at http://bioinformatics.ust.hk/NullDistrAMD.zip. Our analysis result given in Table 2 can be freely reproduced. Following the typical selection procedure of MDR, the four-way interaction (i.e., model ***M***_4_)among SNPs (rs1535891, rs2828151, rs404569, rs380390) is considered as the best one. If the effect of screening and modeling on changing the null distribution is not taken into account, then the hypothesis testing on ***M***_4_ using the null distribution  (see the upper panel of Figure 2 (*d* = 4)) will give a *P*-value of 4.226 × 10^–7^, which is a significant result.

**Table 2:** The experiment result on the AMD data set

Model	SNP name	CV accuracy	CV consistency	χ^2^-value
***M***_1_	rs1329428	0.7246	10/10	27.1480
***M***_2_	rs1329428, rs9299597	0.7086	6/10	38.3007
***M***_3_	rs1535891, rs1329428, rs9299597	0.7833	3/10	55.4297
***M***_4_	rs1535891, rs2828151, rs404569, rs380390	0.7615	10/10	85.2449

**Figure 5:**
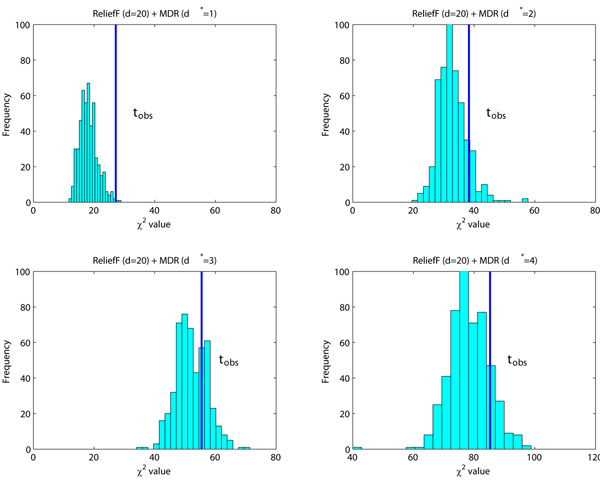
**Null distributions obtained using the permutation test.** We conduct *B* = 500 permutations for the AMD data set, as described in the method section. The *P*-values obtained by the permutation test for models ***M***_1_, ***M***_2_, ***M***_3_, ***M***_4_ are 0.0040, 0.1180, 0.2880 and 0.1480, respectively. Only model ***M***_1_ is significant. The claim of the significance of the high order interaction (rs1535891, rs2828151, rs404569, rs380390) based on Model ***M***_4_ in Table 2 is inappropriate.

The result obtained by using MDR after screening. ReliefF is used to reduce the number of SNPs from ***L*** = 82, 143 to *d* = 20. After that, MDR is applied to the remaining 20 SNPs to search for the best model. The fourth-way interaction model ***M***_4_ is considered as the best model because it gives high CV accuracy and CV consistency. Here CV stands for cross-validation. The χ^2^-value of 85.2449 will correspond to a *P*-value of 4.226 × 10^–7^ against the null distribution  (the upper panel of Figure 2 (*d* = 4)), indicating a significant hypothesis testing result. But the permutation test gives it a *P*-value of 0.1480 (Figure 5), indicating a non-significant result.

Second, we use the permutation test to re-examine the above result. We conducted *B* = 500 permutations to the AMD data set. The details are given in the method section. The result is shown in Figure 5. The *P*-value obtained by the permutation test for model ***M***_4_ is 0.1480, which is far from being significant. More importantly, the permutation result shows that model ***M***_1_ is significant with *P*-value 0.0040. This result is consistent with that in the original paper [37].

Third, we apply testing on the independent data set. The whole data set is partitioned into three groups (49, 49 and 48 individuals, respectively). ReilefF, MDR and hypothesis testing are sequentially applied to them, as described in the method section. Finally, no significant features are reported. This seems different from the result of the permutation test. The reason is that testing on the independent data set has a lower power than the permutation test. The number of samples of the AMD data set is very small (146 individuals). After a nearly equal partition of the data set, only 48 samples can be used in hypothesis testing. Thus no significant results can be detected (see more explanations in the simulation study section). Since the permutation test often has a higher power, the permutation test is preferred when the computational cost is affordable.

## Discussion

### The importance of hypothesis testing in feature assessment

It is important to note that hypothesis testing plays a key role in feature assessment [22,23]. Model selection is a closely related topic, which aims to identify the best model in term of the prediction accuracy [22]. Analytical methods such as Akaike information criterion (AIC), Bayesian information criterion (BIC) can be applied for model selection. Efficient sample re-use methods such as cross-validation and bootstrapping can be applied here as well. However, to statistically quantify the importance of selected features, feature assessment is preferred. Instead of considering prediction accuracy, feature assessment makes use of hypothesis testing to statistically assess the significance of features. To characterize the performance of hypothesis testing, different measures have been defined, e.g., the family wise error rate (FWER) and the false discovery rate (FDR) [35,36]. The Bonferroni correction and the Benjamini-Hochberg method [38] can be used for controlling FWER and FDR, respectively.

As pointed out by Efron [23], the choice of null distribution is critical in hypothesis testing. The empirical null distribution may not match the theoretical null distribution due to reasons such as inappropriate assumptions or correlation across features and samples. This paper shows that screening (e.g, ReliefF) and modeling (e.g, MDR) can also change the null distribution. If their effect is not taken into account in hypothesis testing, the resulting feature assessment will be unreliable.

### Related work

We have shown that inappropriate choice of null distributions will give misleading results of hypothesis testing. One example is that MDR combined with Tuning ReliefF [6,21] will give over-optimistic results. Alternatively, some other methods [25-27], which modify the test statistic but stick to the theoretical null distribution, may produce conservative results. For example, Machini et. al [25] proposed a two-stage method for detecting interactions in genome-wide scale. In the first stage, a single-locus-based test was performed. Those SNPs with significant *P*-values (i.e., smaller than a certain threshold *α*) were selected. The selected set of SNPs was denoted as *I*_1_ (*I*_1_ ⊂ {1,…, ***L***}). In the second stage, for each pair of SNPs *l* and *m* (*l, m ∈ I*_1_*, l ≠ m*), the log likelihood ratio statistic *R*(*l, m*) was calculated for the full interaction model. They defined a new statistic *R*′(*l, m*) = *R*(*l, m*) – (*k_l_* + *k_m_*), where *k_l_* and *k_m_* were the single-locus *χ^2^* values for SNPs *l* and *m*. The significance of this statistic was assessed against  distribution, where *df* = 8 is the degrees of freedom of the full model fitted at the two SNPs. They showed that their method was conservative in term of the false positive rate. In fact, the modified statistic *R*′(*l, m*) is a shrunken version of *R*(*l, m*), but hypothesis testing is performed using the degree of freedom of *R*(*l, m*), where *l*, *m* ∈ {1,…, ***L***}, *l ≠ m*. Thus, this method will be too conservative to detect interesting interactions.

## Conclusion

GWAS have identified many genomic regions associated with complex diseases. However, some previously reported results are based on an inappropriate choice of null distributions, which will produce many false positive results. In this work, we have illustrated that both screening and modeling can change the null distribution used in hypothesis testing. This causes unreliable significance assessment. We have suggested two solutions to address this issue. One is to use the permutation test and another is to use the independent data set for testing. Both solutions can help to appropriately choose null distributions, while the permutation test has a higher power with more computational cost.

## Method

The null distribution is changed after the screening step and the modeling step in the stage-wise procedure. In this section, we suggest two solutions to address this issue. The first solution is to use the permutation test, which uses the re-sampling to generate the reference distribution for hypothesis testing. The second one is to use the independent data set for testing, which reserves the theoretical null distribution.

Suppose we have ***L*** SNPs and *n* samples for an association study. The whole data set ***D*** = [**X**; *Y*] is an (***L*** + 1) × *n* matrix, where we use **X** to denote all SNPs with the *l*-th column *X_l_* corresponding to the *l*-th SNP, and use *Y* to denote the phenotype.

### Permutation test

Since the screening step and the modeling step can change the null distribution, using theoretical null distribution in hypothesis testing may produce biased statistics, which will lead to a high false positive rate. The permutation test can generate the correct null distribution which has accounted for the effects of screening and modeling. This null distribution can be directly used in hypothesis testing. Specifically, the permutation test is done in the following steps:

1. Compute the test statistic based on original data. Apply an efficient screening method to ***D*** and reduce it to ***D***′, where ***D***′ is a (*d* + 1) × *n* matrix collecting the top *d* features. Next, apply modeling methods such as MDR to ***D***′ to identify the best model *f*(X*), where X* has d* features. Then calculate the test statistic of model *f*(X*) based on all samples, denoted as *t_obs_*.

2. Generate *B* independent vectors *Y*_(1)_,…, *Y*_(*B*)_ by randomly permuting the response variable *Y*. Evaluate the permuted statistic *T*_(*b*)_ of the same procedure in Step 1 corresponding to the permuted data set ***D***_(*b*)_ = [**X**; *Y*_(*b*)_], *b* = 1,…,*B*.

3. Calculate the *P*-value as(1)

where *I*(·) is the indicator function.

In theory, a larger number of permutations will produce more accurate estimation. In practice, we typically use between 200 and 1000 permutations due to the high computational cost in the permutation test.

### Testing on the independent data set

**Figure 6:**
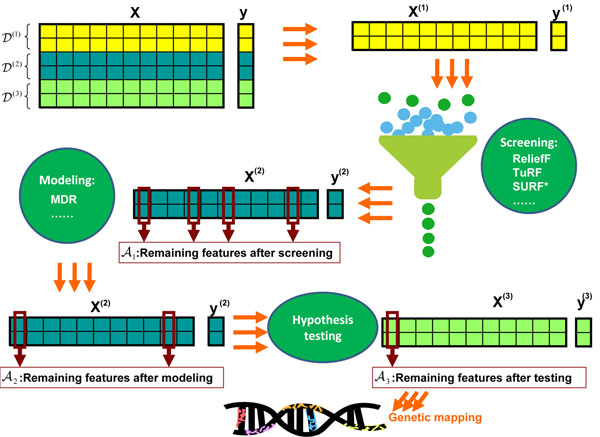
**The procedure of using independent data sets in hypothesis testing.** The whole data set ***D*** is partitioned into three subsets: ***D***^(1)^, ***D***^(2)^ and ***D***^(3)^. A screening method is applied to ***D***^(1)^. After screening, only a subset of features survives, denoted as ***A***_1_. Then modeling methods are applied to ***D***^(2)^, but only involving the features in ***A****_1_*. This modeling process may further select a subset of features from ***A***_1_, denoted as ***A***_2_. Thus, ***A***_2_ ⊂ ***A***_1_. For feature assessment, hypothesis testing is applied to the features in ***A***_2_ using the data set ***D***^(3)^. The correction factor for multiple testing is calculated based on the size of ***A***_2_. After feature assessment, the significant features are collected in ***A***_3_ and ***A***_3_ ⊂ ***A***_2_. They are finally used for genetic mapping.

Screening and modeling pre-use the data that will be used in hypothesis testing. This leads to the change of the null distribution. Using an independent data set in hypothesis testing will avoid the change of the null distribution. It is particularly useful for those methods having analytic null distributions, for example, the likelihood ratio test for logistic regression models. This method consists of the following steps and it is also illustrated in Figure 6.

1. Partition the whole data set ***D*** into three subsets with nearly equal size: ***D***^(1)^, ***D***^(2)^ and ***D***^(3)^.

2. Apply an efficient screening method to ***D***^(1)^ and identify a subset of features, denoted as *A*_1_. Let |***A***_1_| denote the size of ***A***_1_ and we have |***A***_1_| ⊈ ***L***.

3. Apply a modeling method to ***D***^(2)^ by only involving the features in ***A***_1_. The identified features are collected in ***A***_2_ and we have ***A***_2_ ⊂ ***A***_1_.

4. Perform hypothesis test on the features in ***A***_2_ using the data set ***D***^(3)^. The correction factor for multiple testing can be calculated based on |***A***_2_|. The detected significant features are collected in ***A****_3_* and ***A***_3_ ⊂ ***A***_2_. These features are finally used for genetic mapping.

This solution can be applied without sacrificing the running time. However, it has a requirement on the number of samples. A small sample size will degrade its performance. In that situation, the permutation test is a better choice.

## Competing interests

The authors declare that they have no competing interests.

## Authors’ contributions

CY and XW contributed equally to this work. They conducted all experiments. CY, XW and ZH drafted the manuscript together. QY, HX and WY initialized this work. WY finalized the manuscript. All authors read and approved the final manuscript.
